# Developing and validating of Ramathibodi Appendicitis Score (RAMA-AS) for diagnosis of appendicitis in suspected appendicitis patients

**DOI:** 10.1186/s13017-017-0160-3

**Published:** 2017-11-09

**Authors:** Chumpon Wilasrusmee, Boonying Siribumrungwong, Samart Phuwapraisirisan, Napaphat Poprom, Patarawan Woratanarat, Panuwat Lertsithichai, John Attia, Ammarin Thakkinstian

**Affiliations:** 1 Department of Surgery, Faculty of Medicine Ramathibodi Hospital, Mahidol University, Bangkok, Thailand; 2 Section for Clinical Epidemiology and Biostatistics, Faculty of Medicine Ramathibodi Hospital, Mahidol University, Bangkok, Thailand; 3 Department of Surgery, Faculty of Medicine Thammasat University Hospital, Thammasat University, Pathumthani, Thailand; 4 Department of Surgery, Phukhieo Hospital, Chaiyaphum, Thailand; 5 Department of Orthopedics, Faculty of Medicine Ramathibodi Hospital, Mahidol University, Bangkok, Thailand; 6 School of Medicine and Public Health, The University of Newcastle, Newcastle, NSW Australia

**Keywords:** Appendicitis score, Derive phase, Validation phase, Calibration, Discrimination

## Abstract

**Background:**

Diagnosis of appendicitis is still clinically challenging where resources are limited. The purpose of this study was to develop and externally validate Ramathibodi Appendicitis Score (RAMA-AS) in aiding diagnosis of appendicitis.

**Methods:**

A two-phase cross-sectional study (i.e., derivation and validation) was conducted at Ramathibodi Hospital (for derivation) and at Thammasat University Hospital and Chaiyaphum Hospital (for validation). Patients with abdominal pain and suspected of having appendicitis were enrolled. Multiple logistic regression was applied to develop a parsimonious model. Calibration and discrimination performances were assessed. In addition, our RAMA-AS was compared with Alvarado’s score performances using ROC curve analysis.

**Results:**

The RAMA-AS consisted of three domains with seven predictors including symptoms (i.e., progression of pain, aggravation of pain, and migration of pain), signs (i.e., fever and rebound tenderness), and laboratory tests (i.e., white blood cell count (WBC) and neutrophil). The model fitted well with data, and it performed better discrimination than the Alvarado score with C-statistics of 0.842 (95% CI 0.804, 0.881) versus 0.760 (0.710, 0.810). Internal validation by bootstrap yielded Sommer’s D of 0.686 (0.608, 0.763) and C-statistics of 0.848 (0.846, 0.849). The C-statistics of two external validations were 0.853 (0.791, 0.915) and 0.813 (0.736, 0.892) with fair calibrations.

**Conclusion:**

RAMA-AS should be a useful tool for aiding diagnosis of appendicitis with good calibration and discrimination performances.

**Electronic supplementary material:**

The online version of this article (10.1186/s13017-017-0160-3) contains supplementary material, which is available to authorized users.

## Background

Appendicitis is one of the most common causes of acute abdominal pain, with an incidence of 110/100,000 [1]. Although, many attempts have been made to improve the diagnostic accuracy, false negative rates remain common with rates of negative appendectomy of 15 to 26% [2, 3] and perforated appendectomy of 10 to 30% [4].

The critical evaluation of appendicitis should balance between early operation to minimize complicated appendicitis (i.e., perforation, gangrene, and abscess) and a conservative approach reducing unnecessary operation. Several scores had been developed for screening of appendicitis, e.g., Alvarado [5], modified-Alvarado Fenyo [6], Eskelinen [7], etcetera. A systematic review of previous appendicitis scores was conducted to explore their methods used for developments, validations, and performances [8]. Surprisingly, about two-thirds of those studies developed scores based on univariate analysis, and none had evaluated their impacts on health outcome in clinical practice [9]. With poor methodology in previous score developments, we therefore conducted our study, which aimed to develop and externally validate Ramathibodi Appendicitis Score (RAMA-AS).

## Methods

### Study design

The design was a cross-sectional study consisting of derivation and validation phases. Derived data were collected at Ramathibodi Hospital (RH), whereas validated data were collected at Thammasat University Hospital (TH) and Chaiyaphum Hospital (CH) from January 2013 to May 2015. The RH and TH are the Schools of Medicine, whereas CH is a provincial hospital.

The study was conducted and reported according to Transparent Reporting of a Multivariable Prediction Model for Individual Prognosis Or Diagnosis (TRIPOD) [10] and STrengthening the Reporting of OBservational studies in Epidemiology (STROBE) [11]. Consecutive suspected appendicitis patients presenting with abdominal pain were included with following criteria: aged 15–60 years, right side abdominal pain within 7 days, had at least one of the following symptoms (i.e., right lower abdominal pain, migration of abdominal pain, anorexia, nausea, vomiting) and signs (i.e., raised body temperature, right lower quadrant tenderness, guarding, rebound tenderness, and decreased bowel sound), and willing to participate and gave consent. Exclusion criteria were patients who could not give the history of illness, had myocardial infarction or terminal illness, abdominal mass, tumor or malignancy of appendix.

#### Outcome and predictors

The interested outcome was acute appendicitis by histopathological diagnosis for operative patients. For those patients with conservative management, telephone was made to confirm the final diagnosis 6 weeks after visiting.

#### Sample size

As for our literature review, a total of 8–10 variables were potentially included in the final risk prediction score. A simulation study indicated that a number of events per variable of at least 10 to 30 yielded less bias in coefficient estimation of logistic regression [12], which was known as a rule of thumb as per recommendation [13].Using a rule of thumb of at least 20 appendicitis patients per variable required 200 appendicitis patients for 10 variables. The prevalence of appendicitis in our setting was 62% from our pilot study. As a result, 355 patients were needed. Taking into account for missing data of 20%, at least 388 patients were finally required. In addition, an additional 100 subjects (i.e., about 30% of derived subjects) were enrolled from each of the external sites for external validation.

### Statistical analysis

#### Imputation

Multiple imputation was applied to predict missing variables using a simulation-based approach which assumed data were missing at random [14, 15]. A linear truncated regression was applied by regressing missing data on complete data with a number of 20 imputations as per recommendation [16]. Performance of imputation can be assessed using relative variance increase (RVI) and fraction of missing information (FMI). The RVI refers to average relative increase in variances of estimates because of missing variables (i.e., mean of variance of all coefficients from missing data); and as this value closes to 0, missing data reflect less on estimates. The FMI refers to the largest fraction of missing information of coefficient estimates due to missing data. The number of imputations should be roughly estimated based on a rule of thumb, i.e., FMI×100. For instance, if FMI = 0.15, the number of imputations = 0.15 × 100, i.e., at least 15 imputations are required.

#### Derivation

A simple logistic regression analysis was used to screen variables that might associate with appendicitis. Individual variables of 4 domains (i.e., demographic data, clinical symptoms, clinical signs, and laboratory tests) were fitted in a logit model, and a likelihood ratio (LR) test was used to select variables. Variables with *p* values < 0.20 were simultaneously considered in a multivariate logit model. Only significant variables were kept in a parsimonious-model. Goodness of fit was assessed whether the expected (E) or predicted and observed (O) values were close using chi-square Hosmer-Lemeshow test [17]. In addition, a calibration coefficient (O/E) and its 95% confidence interval (CI) were also estimated. The coefficients of the final parsimonious- model were used to create the RAMA-AS. The receiver operating characteristic (ROC) curve, which plotted sensitivity versus 1- specificity, was used to calibrate the score cutoff. Diagnostic parameters (i.e., sensitivity, specificity, likelihood ratio positive (LR+) and negative) were estimated for each distinct value of the scores. The area under ROC, called C-statistic, was estimated, and value close to one reflected higher discrimination of appendicitis from non-appendicitis [18].

#### Validation

##### Internal validation

A bootstrap technique with 450 replications was applied for internal validation of the RAMA-AS [19]. For each bootstrap sample, the RAMA-AS score was calculated and fitted in the logit model. For calibration, the correlation between the observed and expected values of appendicitis was assessed using the Somer’D coefficient for all bootstrap data (called D_boot_) and derived data (called D_org_). Calibration of the model was then assessed by subtracting the D_org_ from the mean D_boot_, and lower value reflected less bias and thus better calibration. Likewise, the original C-statistic was compared to an average C-statistic from the bootstraps for discrimination performance.

##### External validation

Data from the two external hospitals were used to validate the performances of RAMA-AS. Calibration performance was explored as mentioned above. In addition, model re-calibrations were performed by recalibrating intercept (called M1) and overall coefficient (called M2) [20, 21] as follows (see Additional file 1: Table S1: The M1 was constructed by fitting RAMA-AS on appendicitis. The estimated intercept was then used to re-calibrate by adding it up with the original intercept. The estimated coefficient from the M1 was then used to calibrate coefficient by multiplying it with overall coefficients (M2). Four model revisions were additionally performed from the M2 [10, 21–23], (see Additional file 1: Table S1). The M3 was constructed by fitting M2 plus significant predictors by LR test. The M4 was similar to M3 but added significant predictors by stepwise selections. The M5 re-estimated all coefficients of predictors. Finally, the M6 re-selected only significant predictors among all predictors.

Finally, the Alvarado score [5] was compared with the RAMA-AS using ROC curve analysis.

All analyses were performed using STATA version 14 (Stata Corp, College Station, Texas, USA) under mi estimate commands. A *p* value of less than 0.05 was taken as a threshold for statistical significance.

## Results

A total of 396 suspected acute appendicitis patients were enrolled from RH. Among them, 132 patients (33.3%) were male, and mean age and BMI were 36.3 ± 14.6 and 22.8 ± 4.5, respectively. A total of 245/396 (61.8%; 95% CI 56.9%, 66.7%) patients were appendicitis, with a negative appendectomy rate of 4%.

### Imputation

Two variables (i.e., WBC > 10,000 cell/mm^3^ and neutrophil > 75%) contained missing data of 43 (10.9%) and 40 (10.1%), respectively and imputed data were filled in for both variables. Performances of imputation were assessed, and the FMI was < 0.0001 for both variables, indicating 20 imputations were sufficient to fill in missing data, see Additional file 2: Table S2. The diagnostic plot was constructed by comparing missing versus observed values, suggesting no difference between the two values, see Additional file 2: Figure S1.

## Model development

### Derivation

A total of 16 out of 20 predictive variables were suggested from a univariate analysis that they might associate with appendicitis, see Table 1. These included eight symptoms (i.e., first location of pain, migration of pain, onset, progression of pain, right lower quadrant pain at presentation, nausea or vomiting, aggravation of pain by cough or movement, and fever), five signs (i.e., bowel sound, body temperature, tenderness at right lower quadrant of abdomen, rebound tenderness, and guarding), and two laboratory tests (i.e., WBC > 10,000 cell/mm^3^ and neutrophil > 75%).

**Table 1:** Description of patients’ characteristics in appendicitis and non-appendicitis groups

Characteristics	Non-appendicitis *n* = 155	Appendicitis *n* = 241	OR (95% CI)	*p* value
Demographic				
Age (year), mean (SD)	33.8 (11.9)	37.9 (15.9)		< 0.001
Age group				
< 40	99 (63.9)	140 (58.1)	1	0.251
≥ 40	56 (36.1)	101 (41.9)	1.3(0.8–1.9)	
Sex, number, (%)				
Male	39 (25.2)	93 (38.6)	1.9(1.2–2.9)	< 0.001
Female	116 (74.8)	148 (61.4)	1	
BMI, mean (SD)	22.4 (3.9)	22.95 (4.7)		0.230
Symptoms				
First location of pain				
Epigastrium	40 (25.8)	102 (42.3)	2.2(1.4–3.4)	< 0.001
Periumbilical	24 (15.5)	31 (12.9)	1.1(0.6–1.9)	
Other	91 (58.7)	108 (44.8)	1	
Type of pain				
Dull aching, constant	49 (31.6)	82 (34.0)	1.1(0.7–1.7)	0.620
Colicky	106 (68.4)	159 (65.9)	1	
Migration of pain				
Absence	108 (69.7)	111 (46.1)	1	< 0.001
Presence	47 (30.3)	130 (53.9)	2.7(1.8–4.1)	
Onset				
Insidious	120 (77.4)	146 (60.6)	1	< 0.001
Sudden	35 (22.6)	95 (39.4)	2.2(1.4–3.5)	
Progression of pain				
Yes	113 (72.9)	223 (92.5)	4.6(2.5–8.4)	
No	42 (27.1)	18 (7.5)	1	< 0.001
Right lower quadrant pain at presentation				
Yes	140 (90.3)	239 (99.2)	12.8(2.9–56.8)	
No	15 (9.7)	2 (0.8)	1	< 0.001
Time of pain before presentation (hours)				
≤ 48	126 (81.3)	204 (84.7)	1.3(0.7–2.2)	0.382
> 48	29 (18.7)	37 (15.4)	1	
Time of right lower quadrant pain before presentation (hours)				
≤ 12	67 (43.2)	107 (44.4)	1.1(0.7–1.6)	0.820
> 12	88 (56.8)	134 (55.6)	1	
Nausea or vomiting				
Yes	64 (41.3)	141 (58.5)	2.0(1.3–3.0)	
No	91 (58.7)	100 (41.5)	1	< 0.001
Aggravation of pain by cough or movement				
Yes	88 (56.8)	199 (82.6)	3.6(2.3–5.7)	
No	67 (43.2)	42 (17.4)	1	< 0.001
Anorexia				
Yes	118 (76.1)	164 (68.1)	0.7(0.4–1.1)	0.083
No	37 (23.9)	77 (31.9)	1	
Fever				
Yes	135 (87.1)	154 (63.9)	0.3(0.2–0.5)	< 0.001
No	20 (12.9)	87 (36.1)		
Bowel sound				
Increase	20 (12.9)	37 (15.4)	1.4(0.8–2.5)	0.044
Decrease	16 (10.3)	45 (18.7)	2.1(1 .1–3.9)	
Normal	119 (76.8)	159 (65.9)	1	
Body temperature (°C)				
< 37.8	146 (94.2)	176 (73.0)	1	< 0.001
≥ 37.8	9 (5.8)	65 (26.9)	5.9 (2.8–12.4)	
Tenderness at right lower quadrant				
Yes	137 (88.4)	240 (99.6)	31.5 (4.2–238.8)	< 0.001
No	18 (11.6)	1 (0.4)	1	
Rebound tenderness				
Yes	37 (23.9)	155 (64.3)	5.8(3.7–9.1)	< 0.001
No	118 (76.1)	86 (35.7)	1	
Guarding				
Yes	26 (16.8)	82 (34.0)	2.6(1.6–4.2)	< 0.001
No	129 (83.2)	159 (65.9)	1	
Laboratory results				
WBC (cell/mm^3^)				
≤ 10,000	55 (35.5)	26 (10.8)	1	< 0.001
> 10,000 cell/mm^3^	100 (64.5)	215 (89.2)	4.6(2.7–7.7)	
Neutrophil (%)				
≤ 75%	80 (51.6)	54 (22.4)	1	< 0.001
> 75%	75 (48.4)	187 (77.6)	3.7(2.4–5.8)	

These variables were simultaneously included in the logit model, in which only seven variables were remained in the final model. These were three symptoms (i.e., migration of pain, progression of pain, and aggravation of pain by cough or movement), two signs (i.e., body temperature ≥ 37.8 °C and rebound tenderness), two laboratory tests (i.e., WBC > 10,000 cell/mm^3^ and neutrophil > 75%), and odd ratios (OR) and 95% CI were reported, see Table 2. The predictive equation was

$$ \mathit{\ln}\left[P/\right(1-P\Big]=-3.37+(0.80)\mathrm{migration}\  \mathrm{of}\  \mathrm{pain}+(1.04)\mathrm{progression}\  \mathrm{of}\  \mathrm{pain}+(0.78)\mathrm{aggravation}\  \mathrm{of}\  \mathrm{pain}\ \mathrm{by}\ \mathrm{cough}\  \mathrm{or}\  \mathrm{movement}+(1.64)\mathrm{Body}\  \mathrm{temperature}+(1.53)\mathrm{rebound}\  \mathrm{tenderness}+(0.91)\mathrm{white}\  \mathrm{blood}\  \mathrm{cell}+(0.69)\mathrm{neutrophil} $$

**Table 2:** Factor associated with appendicitis: multiple logistic regression analysis

Domain	Parameters	Coefficient	SE	*p* value	OR(95%CI)	Scoring
Symptoms	Progression of pain	1.04	0.4	0.007	2.8 (1.3–5.9)	1.04
	Aggravation of pain by cough or movement	0.78	0.3	0.009	2.2 (1.2–3.8)	0.78
	Migration of pain	0.80	0.3	0.004	2.6 (1.3–3.7)	0.77
Signs	Body temperature ≥ 37.8 °C	1.64	0.5	< 0.001	5.1 (2.1–12.1)	1.64
	Rebound tenderness	1.53	0.3	< 0.001	4.6 (2.7–7.7)	1.53
Lab results	WBC > 10,000 cell/mm^3^	0.91	0.3	0.005	2.6 (1.3–5.0)	0.91
	Neutrophil > 75%	0.69	0.3	0.010	2.3 (1.2–4.1)	0.69
Constant						− 3.37
Total						3.99

*WBC* white blood cell count

#### Model performance

The estimated C-statistic was 0.842 (95% CI 0.804, 0.881), see (Additional file 3: Figure S2), indicating the model well discriminated appendicitis from non-appendicitis. Hosmer-Lemeshow goodness of fit test indicated the model fitted well with the data (chi-square test = 5.64, df = 8, *p* value = 0.687) with the O/E ratio of 0.95 (95% CI 0.83, 1.08).

**Fig. 1:**
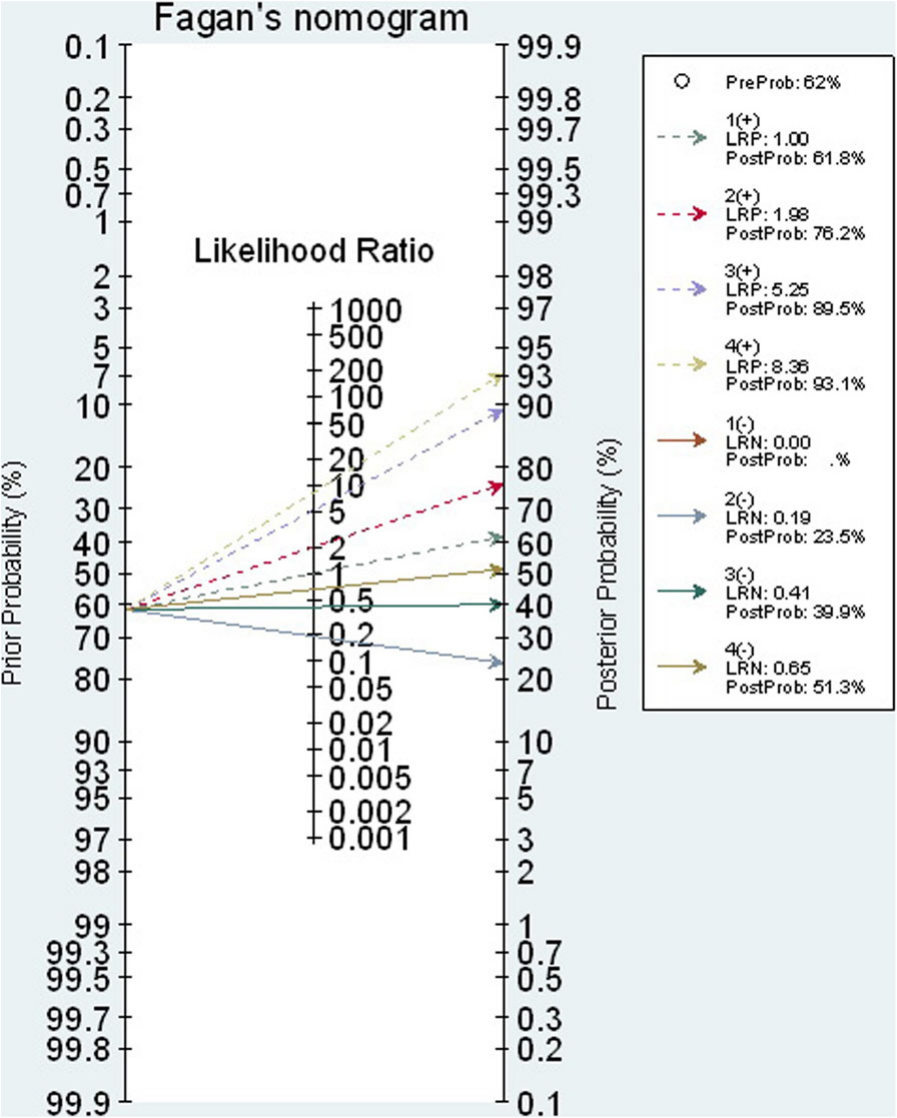
Nomogram plot for RAMA-AS risk stratification

The scoring scheme was constructed using the estimated 7 coefficients, which ranged from − 3.37 to 3.99 with a median of 0.86, see Table 2. The score cutoff was calibrated and stratified into four categories, i.e., very low (score < − 0.64), low (score − 0.64 to 0.84), moderate (score 0.85 to 1.74), and high risk (score > 1.74) groups, see Table 3. The estimated LR+ for these latter three groups were 1.98 (95% CI 1.65, 2.37), 5.25 (95% CI 3.39, 8.13), and 8.36 (95% CI 3.96 to 18.00) when compared to the lowest risk group. The post-test probabilities were 76.0, 89.0, and 93.0% for low, moderate, and high risk groups, respectively (see Fagan plot in Fig. 1).

**Table 3:** Risk stratification and predictive values of a RAMA-AS prediction score

Score	Risk groups	Score development for derivative phase						
		Outcome		% sensitivity (95% CI)	% specificity (95% CI)	LR+ (95% CI)	LR- (95% CI)	Post-positive test odds (%)
		AP	Non-AP					
<− 0.64	Very low	25	85	100.00	0	1.00	0	61.80
− 0.64 to 0.84	Low risk	61	51	89.75 (85.25–93.26)	54.97 (46.67–63.06)	1.98 (1.65–2.37)	0.19 (0.13–0.28)	76.00 (73.00–79.00)
0.85 to 1.74	Moderate	64	12	64.08 (57.73–70.09)	88.08 (81.82–92.78)	5.25 (3.39–8.13)	0.41 (0.34–0.49)	89.00 (85.00–93.00)
> 1.74	High	91	7	37.96 (31.86–44.36)	95.36 (90.68–98.12)	8.36 (3.96–18.00)	0.65 (0.59–0.72)	93.00 (86.00–97.00)

*AP* appendicitis, *LR* likelihood ratio

### Validation

#### Internal validation

The 450 bootstraps yielded estimated D_org_ and D_boot_coefficients of 0.686 and 0.695 (95% CI 0.692, 0.698) for the derivative and bootstrap models, respectively. The bias was only − 0.009 (95% CI − 0.011, − 0.007), suggesting good calibration. The bootstrap C-statistics was 0.848 (95% CI 0.846, 0.849), with a bias of − 0.005 (95% CI − 0.006, − 0.004).

#### External validation

A total of 330 patients with suspected acute appendicitis (152 and 178 from TH and CH, respectively) were used to externally validate the RAMA-AS. Their characteristics were described in Table 4.

**Table 4:** Describe characteristics of patients from derivation and external validation data

Characteristics	RA (*n* = 396)	TS (*n* = 152)	CP (*n* = 178)
Mean age (SD), years	36.3(14.6)	35.6(16 .9)	42.9(16.8)
Men	132 (35.8%)	40 (26.4%)	71 (39.9%)
Symptoms			
Progression of pain	336 (84.8%)	98 (64.5%)	147 (82.6%)
Aggravation of pain	287 (72.5%)	78 (51.4%)	104 (58.4%)
Migration of pain	177 (44.7%)	73 (48.0%)	125 (70.2%)
Signs			
Body temperature ≥ 37.8 °C	74 (18.7%)	30 (19.7%)	67 (37.6%)
Rebound tenderness	192 (48.5%)	65 (42.8%)	127 (71.3%)
Laboratory			
WBC	315 (79.6%)	125 (82.2%)	141 (79.2%)
Neutrophil	262 (66.2%)	115 (75.7%)	124 (69.7%)
Prevalence of appendicitis	245/396 (61.8%)	74/152 (48.7%)	137/178 (76.9%)

*CP* Chaiyaphum Hospital, *RA* Ramathibodi Hospital, *TS* Thammasat University Hospital

##### Thammasat University Hospital

Comparing with RH, prevalence of appendicitis was much lower in TH, i.e., 48.7 vs 61.8, %, but the mean age was quite similar (35.6 vs 36.3 years), although the male percentage was much lower (26.4 vs 35.8%), see Table 4. Among seven predictors, distributions of rebound tenderness (42.8 vs 48.5%), progression of pain (64.5 vs 84.8%), and aggravation of pain (51.4 vs 72.5%) were little to much lower, but migration of pain (48.0 vs 44.7%), body temperature (19.7 vs 18.7%) and WBC > 10,000 cell/mm^3^ (82.2 vs 79.6%) and neutrophil > 75% (75.7 vs 66 .2%) were little to much higher differences. These variables were also described by appendicitis groups, indicating higher prevalence for all symptoms and signs, but not for laboratory tests, see Additional file 1: Table S3.

The estimated RAMA-AS, which ranged from − 3.4 to 4.0, seemed to work well in TH with the estimated O/E ratio of 1.005 (95% CI 0.784, 1.225; Hosmer-Lemeshow = 8.219, (df = 4), *p* = 0.084). However, the calibration plot showed the predicted risk deviated from the reference line (see Additional file 4: Figure S3-A), i.e., under-estimated risk for lower score and over-estimated risk for higher scores. The intercept and overall coefficients were then calibrated (see Additional file 1: Table S4), and calibration plots were constructed (see Additional file 4: Figure S3-B-C) which suggested no improvement of calibrations.

Revision M3 models by LR test indicated that migration of pain, progression of pain, body temperature, WBC, and neutrophil were significant predictors, see Additional file 1: Table S4. Comparing coefficients of M3 versus coefficients of the original RH model in Table 2, coefficients of body temperature, WBC, and neutrophil were changed from positive to negative coefficients, whereas coefficients of the rest of the predictors increased. Only migration of pain, progression of pain, and rebound tenderness were significant by stepwise selection for M4. Of these, progression of pain and rebound tenderness were much lower but migration of pain was higher than in RH, see Table 2 and Additional file 1: Table S4.

Calibration coefficients of these models were estimated, which resulted in the O/E ratio for revision M3 model and M4 of 0.940 (95% CI 0.729, 1.150; Hosmer-Lemeshow = 2.683, df = 4, *p* = 0.612) and 1.006 (95% CI 0.743, 1.269; Hosmer-Lemeshow = 5.00, df = 4, *p* = 0.287), respectively, which were much improved when compared to the M0. Calibration plots also showed better fits with the reference lines when compared to the M0, see Additional file 4: Figure S3 A, D-E. The M5 which entered all seven predictors or stepwise selection in M6 yielded similar results as M4, in which only three predictors (i.e., migration of pain, progression of pain, and rebound tenderness) were significant. The O/E ratios were 0.870 (0.578, 1.612) and 0.947 (95% CI 0.684, 1.209) and calibration plots showed better fit than M0, see Additional file 4: Figure S3 F-G.

C-statistics were estimated for all models, see Additional file 1: Table S5. These suggested that the M0 could well discriminate appendicitis from non-appendicitis with the C-statistics of 0.853 (95% CI 0.790, 0.915), and they were little improved for M3, M4, and M6, but not for M5, see Additional file 1: Table S5.

##### Chaiyaphum Hospital

Comparing with RH (see Table 4), prevalence of appendicitis in CH was much higher (76.9 vs 61.8%), and mean age (42.9 vs 36.3 years) and male percentage were higher (39.9 vs 35.8%). Migration of pain (70.2 vs 44.7%), body temperature (37.6% vs 18.7%), and rebound tenderness (71.3 vs 48.5%) were more present, but aggravation of pain was much lower (58.4 vs 72.5%), whereas progression of pain (82.6 vs 84.8%), WBC > 10,000 cell/mm^3^ (76.9 vs 79.6%) and neutrophil (63.5 vs 66.2%) were little lower than RH. Distribution of these predictors between appendicitis groups were described, and all except neutrophil were more prevalent in appendicitis than non-appendicitis groups, in Additional file 1: Table S3.

A median RAMA-AS was 1.6 (− 3.4, 4.0) with O/E ratio of 0.996 (95% CI 0.695, 1.333; Hosmer-Lemeshow = 6.640 (df = 4), *p* = 0.156), see Additional file 1: Table S5. Calibration models were constructed (see Additional file 1: Table S4) and plotted (see Additional file 5: Figure S4 A-G). These suggested that the M0 still deviated from the reference line particularly for low and high scores. M1 and M2 did not improve calibrations when compared to the original M0. Among revision models, M3-M6, M3-M4, and M6 were improved in calibrations, particularly the M6 was the best with O/E ratios of 1.021 (95% CI 0.905, 1.186), whereas the calibration plot of M5 showed quite poor performance.

The M0’s discrimination performance was good, although it was lower than the original model (C-statistic = 0.813; 0.736, 0.892). The C-statistics for M3 to M6 were a bit higher than M0, see Additional file 1: Table S5.

### Comparison of RAMA-AS and previous score

**Fig. 2:**
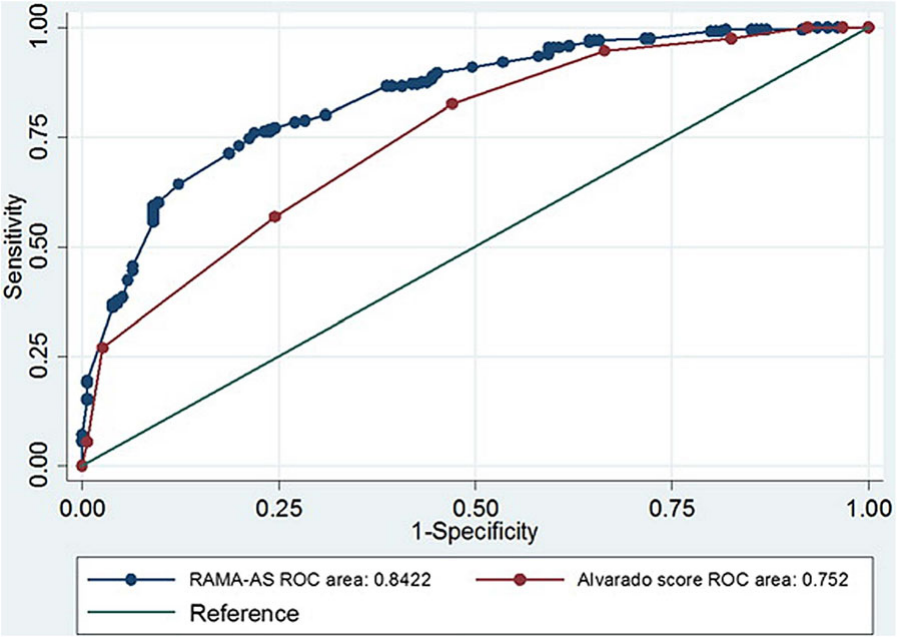
Receiver operating characteristic (ROC) curves presenting the comparison of Alvarado’score (dash line) and RAMA-AS (solid line)

Alvarado scores was calculated which ranged of 2 to 10 (mean = 7.04). The C-statistics was 0.752 (95% CI 0.710, 0.800) which was statistically lower than RAMA-AS (*p* value of < 0.001, see Fig. 2).

## Discussion

We developed and internally and externally validated a RAMA-AS, for classifying very low, low, moderate, and high risk of having appendicitis. Predictive domains including three symptoms, two signs, and two laboratory tests were included. Internal validation showed the RAMA-AS performed well for both calibration and discrimination. The external validation showed fair calibrations and good discrimination with the O/E ratios of 1.01 (0.78, 1.23) and 0.996 (0.659, 1.333), with the C-statistics of 0.853 (95% CI 0.791, 0.915) and 0.817 (95% CI 0.736, 0.892), respectively.

Although most predictors of clinical signs, symptoms, and laboratory tests used in the RAMA-AS were similar to the Alvarado score, which was the most commonly used in prospective studies [6, 24–29], our performances were better. This might be due to difference in weighting or scoring for each predictor, distribution of predictors, and also prevalence of appendicitis itself. Our score was derived based on proper model construction, following the recommendation by TRIPOD [10], and let the data suggest proper weighting. Our finding was consistent to the appendicitis inflammatory response (AIR) [30], developed in 2008, which externally performed better than the Alvarado score. This score did not consider WBC and neutrophil, but instead included leukocyte and CRP in the model [30, 31], in which the CRP may be not a routine laboratory test in some developing countries. Thus, it is not easily applied in the setting where resources are limited. Our RAMA-AS and also these scores could rule out well, but not rule in as per WSES Jarusalem guidelines [30], so high risk score may need confirmation by CT scan [31].

Calibration performance of RAMA-AS was fair in both external data sets. This could be explained as follows: first, prevalence of appendicitis in the derived RH and validated TH and CH’s were reasonably different, i.e., 61.8 vs 48.7 vs 76.9%, respectively. Therefore, the original model over-estimated risk of appendicitis in TH, but under estimated risk in CH. We then re-calibrated the intercept in M1 models by minus and plus the original intercept (i.e., baseline risk) with estimated intercepts for TH and CH, respectively. These models were still not well calibrated, we thus moved further to recalibrate overall coefficient (M2), but this did not much improve. Differences in distributions of predictors between appendicitis groups across data sources may also play a role. For instance, all symptoms and signs were more present in appendicitis than in non-appendicitis groups for both external hospitals, but not for WBC and neutrophil. The revisions of models showed much improvement, which could be M4 or M6 for both TH and CH. Only two symptoms and one sign contributed in predictions for both hospitals, therefore, the predictive score containing only three symptoms (migration of pain, progression of pain, aggravation of pain) and one sign (rebound tenderness) without laboratory test is proposed. Its performances in calibration and discrimination was very much similar to M6 (data were not shown). Although the RAMA-AS did not perform well in the external data when compared to the derived data, it could still well discriminate appendicitis from non-appendicitis in provincial setting (CH) and School of Medicine setting (TH).

### Using the RAMA-AS in practice

Our RAMA-AS should be applied in general hospitals where resources are limited. Data of seven variables can be collected from physical examination, interview, and CBC test. Applying the RAMA-AS is easy by inputting data in the equation. Probability of appendicitis is then estimated for each risk stratification using Fagan nomogram. In addition, the score can be straight forwardly classified as very low (score < − 0.64), low (score − 0.64 to 0.84), moderate (score 0.85 to 1.74), and high risk (score > 1.74) of having appendicitis. As for the ROC analysis, these cut-off thresholds were objectively selected based on LR+ (i.e., sensitivity/(1- specificity)), which had less bias than subjective selection [32]. Although our score could well discriminate appendicitis from non-appendicitis as for the C-statistics, clinical findings should also be incorporated for further decision making. Imaging investigation may be needed for moderate to high scores [31].

Counting number of positive of signs, symptoms, and laboratory results can be also applied. For instance, low risk appendicitis if having only positive for all items of signs, symptom, or laboratory tests; 1 positive item for each of 3 domains; 2 positive items among 3 domains (i.e., 1 symptom and sign, 1 symptom and laboratory test, 1 sign and 1 laboratory test); 3 symptoms with 1 laboratory test without sign; 3 symptoms plus one sign without laboratory test. The post-test probability would be 76.0%, so out-patient observation is recommended. The moderate risk requires three symptoms plus one sign of body temperature ≥ 37.8 °C, or three symptoms plus two laboratory tests without any sign. The post-test probability is from 85.0 to 93.0% for moderate risks, so other investigations such as ultrasound or CT scan may be needed for these patients.

The high risk group requires all symptoms and signs, or all symptoms plus one sign and laboratory test, all symptoms plus two signs plus any of laboratory test, or three symptoms plus two laboratory tests plus any of the signs. The post-test probability is about 93.0% and thus surgical treatment should be performed for high risk patients.

Our study has some strengths. We followed the recommendations for developing risk prediction score by Altman et al. [33] and TRIPOD [10]. We developed and both internally and externally validated the scores using prospective data collections. Imputation of missing data was applied, even though it occurred only on a few variables, which should yield better performances of risk prediction model than analysis of complete case only [34]. The RAMA-AS showed good performances for both calibration and discrimination in the derived setting, although one external setting had lower discrimination performance.

However, some limitations could not be avoided. The study was conducted at tertiary hospitals where the appendicitis prevalence was high. The RAMA-AS should be further validated in different populations and settings. In order to improve generalizability, big electronic health data or individual patient meta-analysis should be conducted [35]. Clinical impact of the RAMA-AS should be also further assessed. For instance, applying the score in a routine clinical practice, which will let us know whether our score, can still well rule out and rule in suspected patients with and without appendicitis. These suspected patients may be only observed or treated with operation or even non-operative treatment such as antibiotics. Previous cohort study showed long-term success and safety of antibiotics in suspected appendicitis [36]. However, this evidence was from observational study, which was prone to selection bias. Individual randomized controlled trial with appropriate methods should be conducted to test if non-operative treatment is non-inferior to operation [37].

## Conclusions

Appendicitis is one of the most important clinical causes among acute abdominal pain. Several scoring systems had been developed for screening of appendicitis. Surprisingly, about two-thirds of studies developed prediction scores based on univariate analysis without applying statistical modeling. We have developed and internally/externally validated a clinical prediction score, called RAMA-AS, to classify risk of having appendicitis. The RAMA-AS showed good internal but fair external calibration, and it well discriminated for both internal and external validations. The RAMA-AS performed better than the Alvarado system (i.e., C-statistics 0.840 VS 0.710), which can suggest whether patients can be observed as out-patients, need further investigation or admit for appendectomy.

## Additional files

Additional file 1: Table S1. Re-calibration and revision of models for external validations. **Table S2.** Report number of missing data. **Table S3**. Distributions of predictors by appendicitis groups and developed/validated data. **Table S4**. Estimation of intercept and coefficients for external validations using different update models. **Table S5**. Estimations of calibration coefficients and C-statistics for external validations using different re-calibration and revision methods. (DOCX 57 kb)

Additional file 2: Figure S1. Diagnosis plot between missing and observed values: A) WBC, B) Neutrophil. (PDF 157 kb)

Additional file 3: Figure S2. Receiver operating characteristic (ROC) curves of RAMA-AS for diagnosis of appendicitis. (PDF 153 kb)

Additional file 4: Figure S3. Calibration plots for external validations at Thammasat University Hospital using different update methods. (ZIP 298 kb)

Additional file 5: Figure S4. Calibration plots for external validations at Chaiyapum Hospital using different update methods. (ZIP 298 kb)

## Abbreviations

CH: Chaiyaphum Hospital
CI: Confidence interval
E: Expected relative variance increase
FMI: Fraction of missing information
LR: Likelihood ratio
O: Observed
RAMA-AS: Ramathibodi Appendicitis Score
RH: Ramathibodi Hospital
ROC: receiver operating characteristic
RVI: Relative variance increase
STOBE: STrengthening the Reporting of OBservational studies in Epidemiology
TH: Thammasat University Hospital
TRIPOD: Transparent Reporting of a Multivariable Prediction Model for Individual Prognosis Or Diagnosis
