# Supplementary material for: Developing and validating of Ramathibodi Appendicitis Score (RAMA-AS) for diagnosis of appendicitis in suspected appendicitis patients
Source: World J Emerg Surg. 2017 Nov 9;12:49. doi: 10.1186/s13017-017-0160-3 (PMC5679324; doi:10.1186/s13017-017-0160-3)
Supplement: Supplementary file 1 — Re-calibration and revision of models for external validations. Table S2. Report number of missing data. Table S3. Distributions of predictors by appendicitis groups and developed/validated data. Table S4. Estimation of intercept and coefficients for external validations using different update models. Table S5. Estimations of calibration coefficients and C-statistics for external validations using different re-calibration and revision methods. (DOCX 57 kb) [file 13017_2017_160_MOESM1_ESM.docx]

**Additional Tables**

Table S1. Re-calibration and revision of models for external validations

Table S2. Report number of missing data

Table S3. Distributions of predictors by appendicitis groups and developed/validated data

Table S4 Estimation of intercept and coefficients for external validations using different update models

Table S5. Estimations of calibration coefficients and C-statistics for external validations using different re-calibration and revision methods

Table S1. Re-calibration and revision of models for external validations

| Type of update model | Thammasat | Chaiyaphum |
| --- | --- | --- |
| M_0_: Original model |  |  |
| $\hat{\alpha}_{0}$ |  |  |
| M_1_: Re-calibrate intercept |  |  |
| α |  |  |
| M_2_: Re-calibration |  |  |
| α |  |  |
| β_overall_ |  |  |
| M_3_: Revision M_2_+γ_i_X_i_ |  |  |
| α |  |  |
| β_overall_ |  |  |
| Likelihood ratio test |  |  |
| M_4_: Revision M_2_+γ_i_X_i_ |  |  |
| α |  |  |
| β_overall_ |  |  |
| Stepwise selection |  |  |
| M_5_: |  |  |
| Enter all predictors |  |  |
| M_6_: |  |  |
| Stepwise selection |  |  |

Table S2. Report number of missing data

| Missing Variables | Percent | Observed | Imputed | FMI | RVI |
| --- | --- | --- | --- | --- | --- |
| WBC | 10.86 | 353 | 43 | <0.0001 | <0.0001 |
| Neutrophil | 10.10 | 356 | 40 | <0.0001 | <0.0001 |

FMI, fraction of missing information; RVI, relative variance increase

**Table S3.** Distributions of predictors by appendicitis groups and developed/validated data

| Predictors | Ramathibodi Hospital | | Thammasat University Hospital | | Chaiyaphum Hospital | |
| --- | --- | --- | --- | --- | --- | --- |
|  | AP | Non-AP | AP | Non-AP | AP | Non-AP |
| Progression of pain  Yes | 223(92.5) | 113(72.9) | 67(90.5) | 31(39.7) | 123(87.9) | 11(61.1) |
| No | 18(7.5) | 42(27.1) | 7(9.5) | 47(60.3) | 17(12.1) | 7(38.9) |
| Aggravation of pain  Yes | 199(82.6) | 88(56.8) | 55(74.3) | 23(29.5) | 96(68.6) | 9(50.0) |
| No | 42(17.4) | 67(43.2) | 19(25.7) | 55(70.5) | 44(31.4) | 9(50.0) |
| Migration of pain  Yes | 130(53.9) | 47(30.3) | 55(74.3) | 18(23.1) | 105(75.0) | 9(50.0) |
| No | 111(46.1) | 108(69.7) | 19(25.68) | 60(76.9) | 35(25.0) | 9(50.0) |
| Body temperature  ≥37.8 °C | 65(26.9) | 9(5.8) | 21(28.4) | 9(11.5) | 52(37.1) | 1(5.6) |
| < 37.8 °C | 176(73.0) | 146(94.2) | 53(71.6) | 69(88.5) | 88(62.9) | 17(94.4) |
| Rebound tenderness  Yes | 155(64.3) | 37(23.9) | 48(64.9) | 17(21.8) | 119(85.0) | 6(33.3) |
| No | 86(35.7) | 118(76.1) | 26(35.1) | 61(78.2) | 21(15.0) | 12(66.7) |
| WBC (cell/mm^3^)  >10,000 | 215(89.2) | 100(64.5) | 58(78.4) | 67(85.9) | 122(87.1) | 13(72.2) |
| ≤10,000 | 26(10.8) | 55(35.5) | 16(21.6) | 11(14.1) | 18(12.9) | 5(27.8) |
| Neutrophil (%)  >75 | 187(77.6) | 75(48.4) | 54(72.9) | 61(78.2) | 102(72.9) | 13(72.2) |
| ≤75 | 54(22.4) | 80(51.6) | 20(27.0) | 17(21.8) | 38(27.1) | 5(27.8) |

Table S4 Estimation of intercept and coefficients for external validations using different update models

| Type of update model | Thammasat | Chaiyaphum |
| --- | --- | --- |
| M_0_: Original model |  |  |
| α=-3.374 | 0.376 | 0.501 |
| M1: Re-calibration |  |  |
| Α | -3.374-0.376 | -3.374+(0.501) |
| M2: Re-calibration |  |  |
| α | -3.374-0.376 | -3.374+(0.501) |
| β_overall_ | 0.929 | 0.798 |
| M3: Revision M2+γ_i_X_i_ |  |  |
| α | -3.374-0.376 | -3.374+(0.501) |
| β_overall_ | 0.929 | 0.798 |
| Migration of pain | 1.284 (0.004) | 0.0391 (0.930) |
| Progression of pain  Aggravation of pain | 1.138 (0.046)  0.426 (0.378) | -0.490 (0.391)  0.629 (0.184) |
| Body temperature | -1.332 (0.033) | -1.339 (0.024) |
| Rebound tenderness | 0.454 (0.332) | 1.937 (<0.001) |
| WBC | -1.353 (0.017) | -0.333 (0.519) |
| Neutrophil | -1.236 (0.017) | -1.275 (0.018) |
| M4: |  |  |
| α | -3.374-0.376 | -3.374+(0.501) |
| β_overall_ | 0.929 | 0.798 |
| Migration of pain | 1.836 (<0.001) | 0.957 (0.043) |
| Progression of pain  Aggravation of pain | 1.768 (0.001) | 1.128 (0.016) |
| Rebound tenderness | 1.817 (<0.001) | 2.619 (<0.001) |
| M5: |  |  |
| α | -3.189 | -1.965 |
| Migration of pain | 1.816 (<0.001) | 1.011 (0.041) |
| Progression of pain | 1.539 (0.006) | 0.197 (0.716) |
| Aggravation of pain | 0.512 (0.286) | 0.988 (0.043) |
| Body temperature | 0.485 (0.378) | 0.405 (0.431) |
| Rebound tenderness | 1.720 (<0.001) | 2.566 (<0.001) |
| WBC | 0.345 (0.615) | 1.129 (0.061) |
| Neutrophil | -0.282 (0.658) | -0.757 (0.221) |
| M6: |  |  |
| α | -2.959 | -1.430 |
| Migration of pain | 1.836 (<0.001) | 0.957 (0.043) |
| Progression of pain | 1.768 (0.001) |  |
| Aggravation of pain |  | 1.128 (0.016) |
| Rebound tenderness | 1.817 (<0.001) | 2.619 (<0.001) |

Table S5. Estimations of calibration coefficients and C-statistics for external validations using different re-calibration and revision methods

| Model | Thammasat | | | Chaiyaphum | | |
| --- | --- | --- | --- | --- | --- | --- |
|  | GoF test  P value | O/E (95%CI) | C-statistics (95% CI) | GoF  P value | O/E  (95%CI) | C-statistics  (95% CI) |
| M0 | 0.084 | 1.01  (0.78, 1.23) | 0.853  (0.791, 0.915) | 0.156 | 0.996  (0.659,1.333) | 0.813  (0.736, 0.892) |
| M1 | 0.084 | 1.00  (0.78, 1.23) | 0.853  (0.791, 0.915) | 0.156 | 0.996  (0.659, 1.333) | 0.813  (0.736, 0.892) |
| M2 | 0.084 | 1.00  (0.78, 1.23) | 0.853  (0.791, 0.915) | 0.156 | 0.996  (0.659, 1.333) | 0.813  (0.736, 0.892) |
| M3 | 0.612 | 0.94  (0.73, 1.15) | 0.877  (0.823, 0.932) | 0.261 | 1.083  (0.734,1.434) | 0.854  (0.777, 0.931) |
| M4 | 0.287 | 1.01  (0.74, 1.27) | 0.881  (0.828, 0.935) | 0.239 | 1.035  (0.651, 1.419) | 0.857  (0.788, 0.926) |
| M5 | 0.270 | 0.87  (0.58, 1.61) | 0.884  (0.832, 0.936) | 0.279 | 0.905  (0.622, 1.186) | 0.873  (0.809, 0.938) |
| M6 | 0.354 | 0.95  (0.68, 1.21) | 0.872  (0.817, 0.926) | 0.967 | 1.021  (0.947, 1.094) | 0.860  (0.790, 0.930) |

GoF, goodness of fit
