# Supplementary figures and images for: Developing and validating of Ramathibodi Appendicitis Score (RAMA-AS) for diagnosis of appendicitis in suspected appendicitis patients
Source: World J Emerg Surg. 2017 Nov 9;12:49. doi: 10.1186/s13017-017-0160-3 (PMC5679324; doi:10.1186/s13017-017-0160-3)

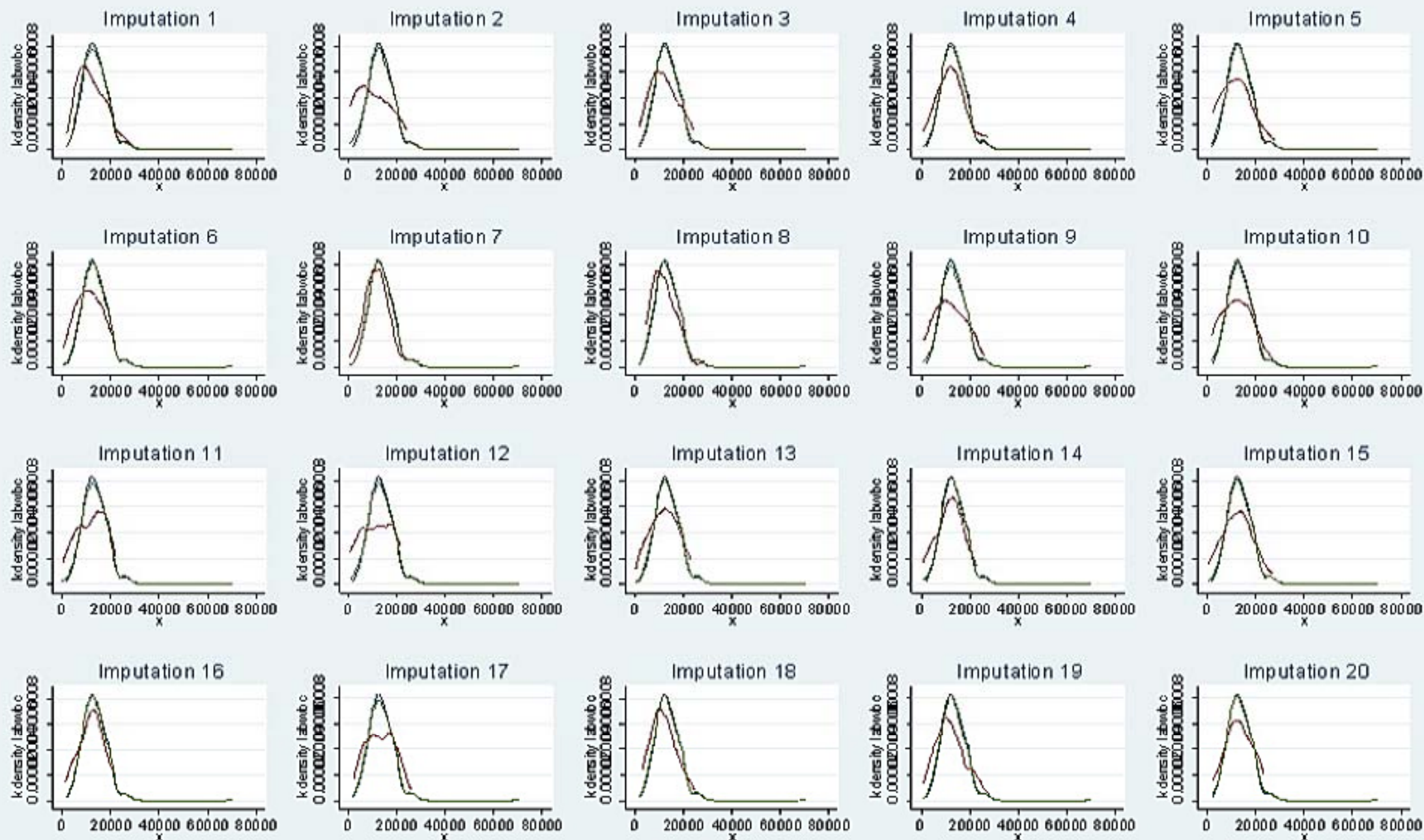

— Observed    — Imputed    — Completed

Supplement: Supplementary file 2 — Diagnosis plot between missing and observed values: A) WBC, B) Neutrophil. (PDF 157 kb) [file 13017_2017_160_MOESM2_ESM.pdf]

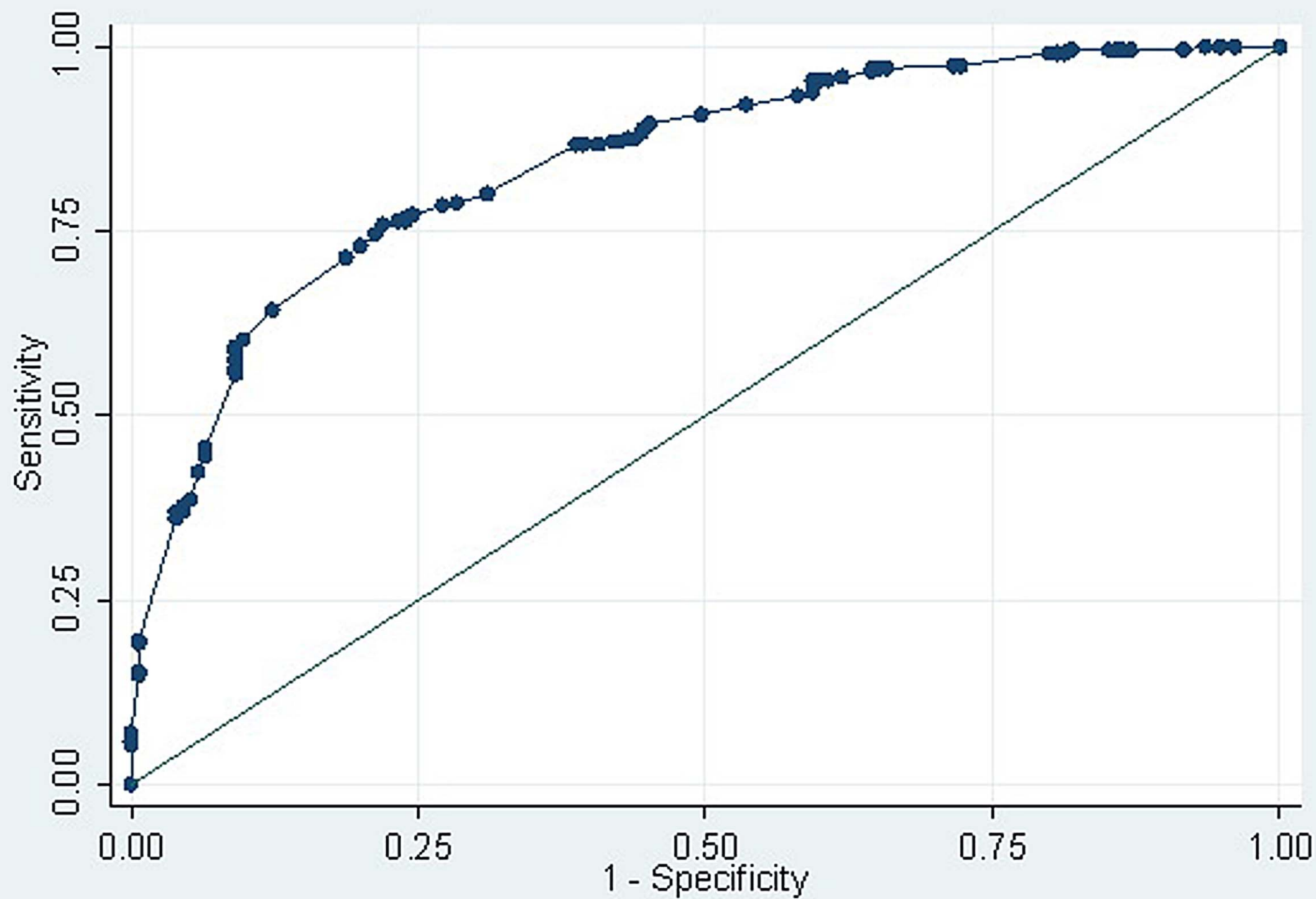

Area under ROC curve = 0.8422

Supplement: Supplementary file 3 — Receiver operating characteristic (ROC) curves of RAMA-AS for diagnosis of appendicitis. (PDF 153 kb) [file 13017_2017_160_MOESM3_ESM.pdf]

A) Original model M0

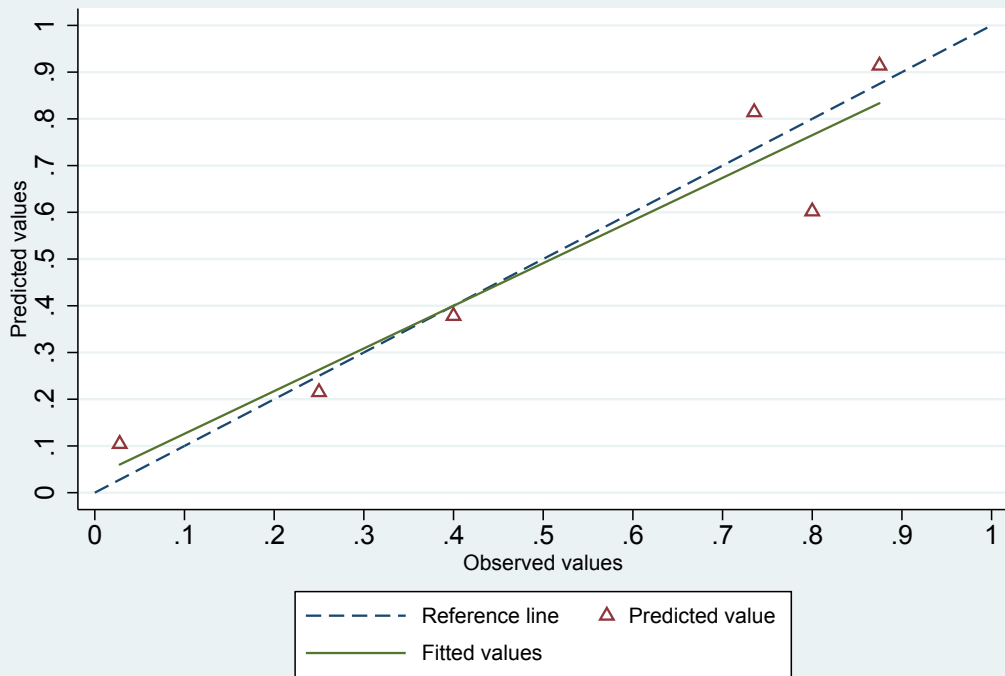

Supplement: Supplementary file 4 — Calibration plots for external validations at Thammasat University Hospital using different update methods. (ZIP 298 kb) [file 13017_2017_160_MOESM4_ESM.zip › Additional fig 3 A-M0.pdf]

B) Re-calibration intercept M1

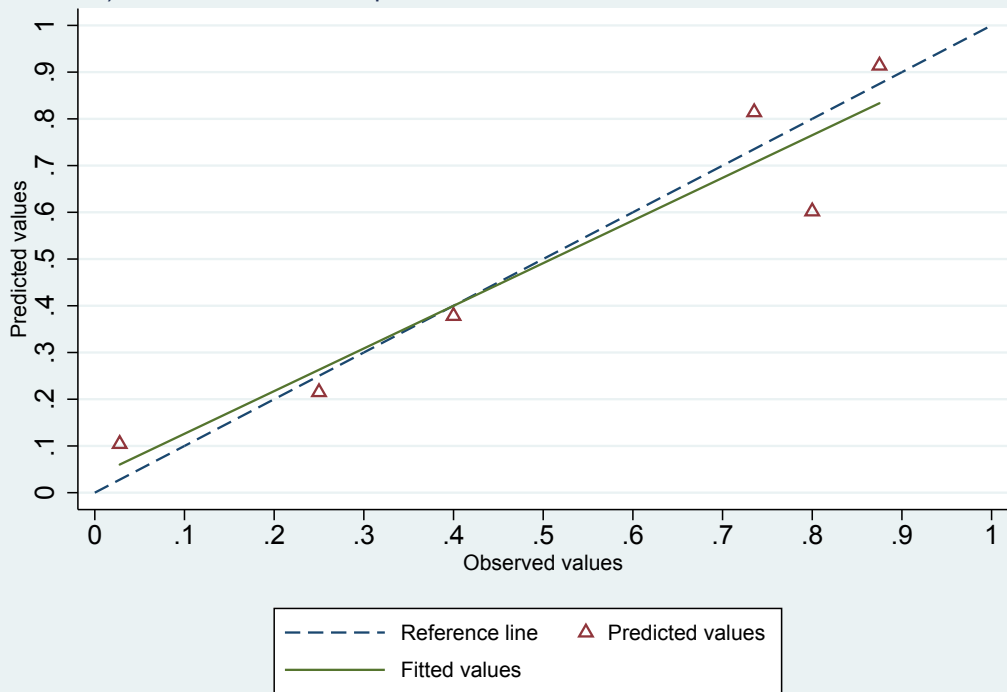

Supplement: Supplementary file 4 — Calibration plots for external validations at Thammasat University Hospital using different update methods. (ZIP 298 kb) [file 13017_2017_160_MOESM4_ESM.zip › Additional fig 3 B-M1.pdf]

C) Re-calibration intercept and coefficient M2

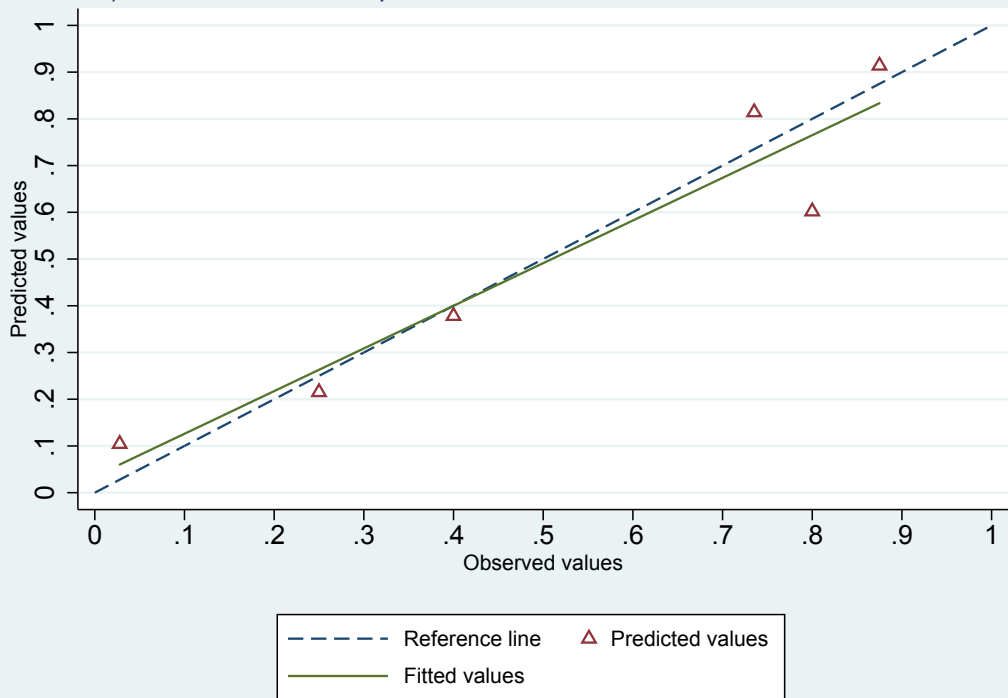

Supplement: Supplementary file 4 — Calibration plots for external validations at Thammasat University Hospital using different update methods. (ZIP 298 kb) [file 13017_2017_160_MOESM4_ESM.zip › Additional fig 3 C-M2.pdf]

D) Revision model M3

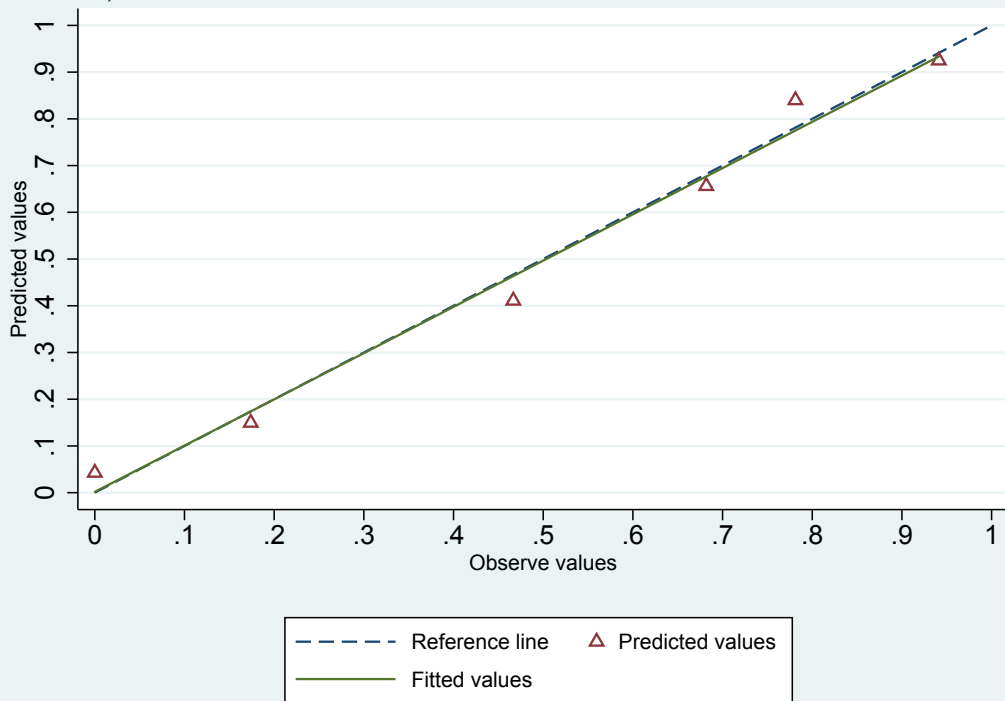

Supplement: Supplementary file 4 — Calibration plots for external validations at Thammasat University Hospital using different update methods. (ZIP 298 kb) [file 13017_2017_160_MOESM4_ESM.zip › Additional fig 3 D-M3.pdf]

E) Revision model M4

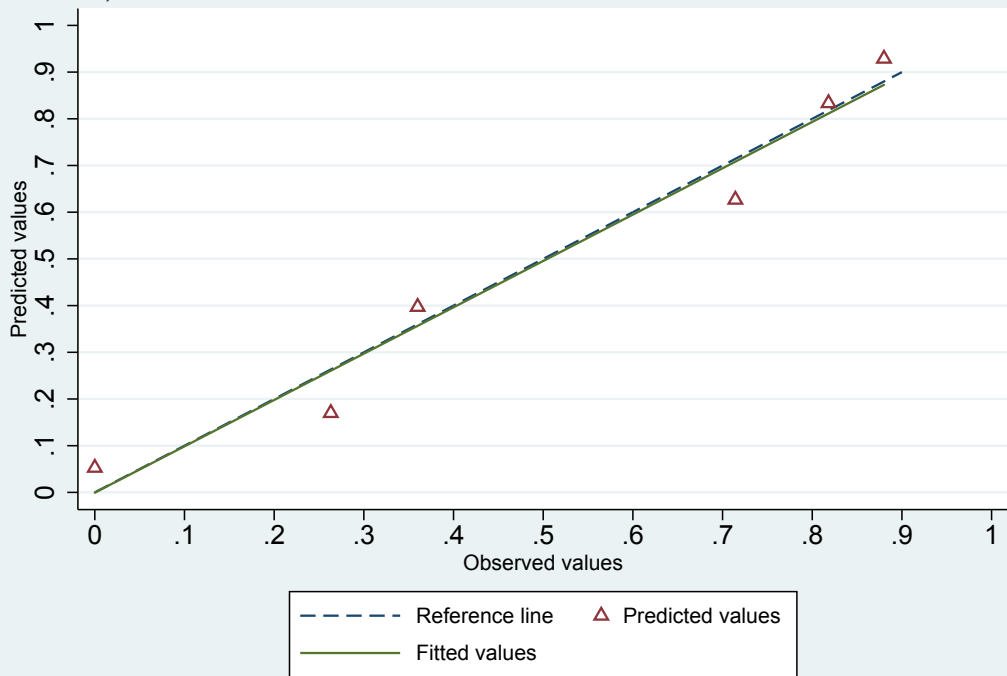

Supplement: Supplementary file 4 — Calibration plots for external validations at Thammasat University Hospital using different update methods. (ZIP 298 kb) [file 13017_2017_160_MOESM4_ESM.zip › Additional fig 3 E-M4.pdf]

F) Revision model M5

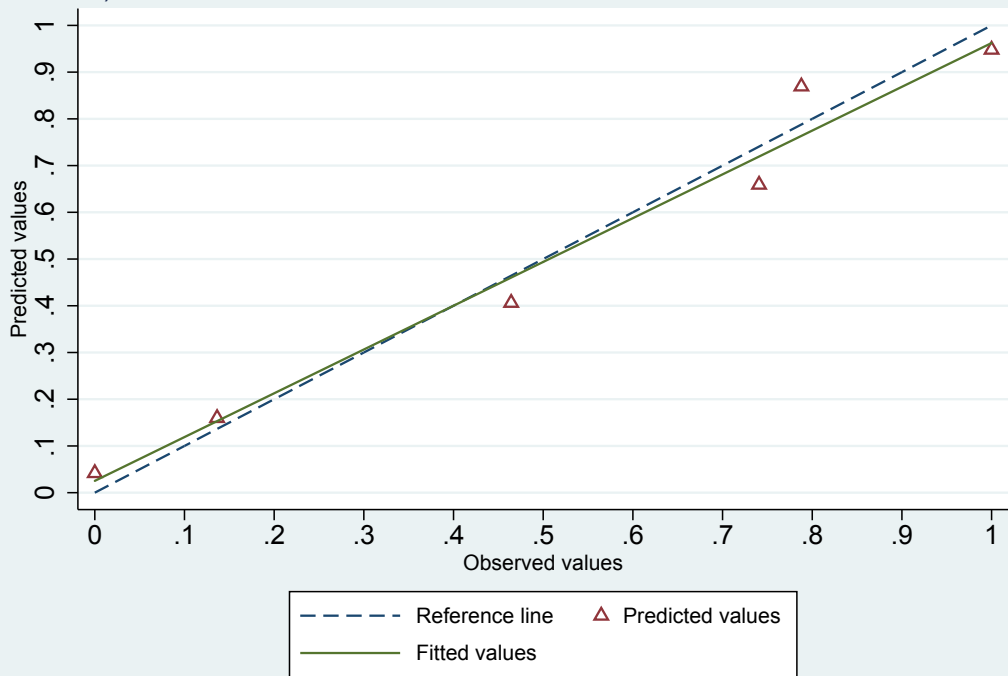

Supplement: Supplementary file 4 — Calibration plots for external validations at Thammasat University Hospital using different update methods. (ZIP 298 kb) [file 13017_2017_160_MOESM4_ESM.zip › Additional fig 3 F-M5.pdf]

G) Revision model M6

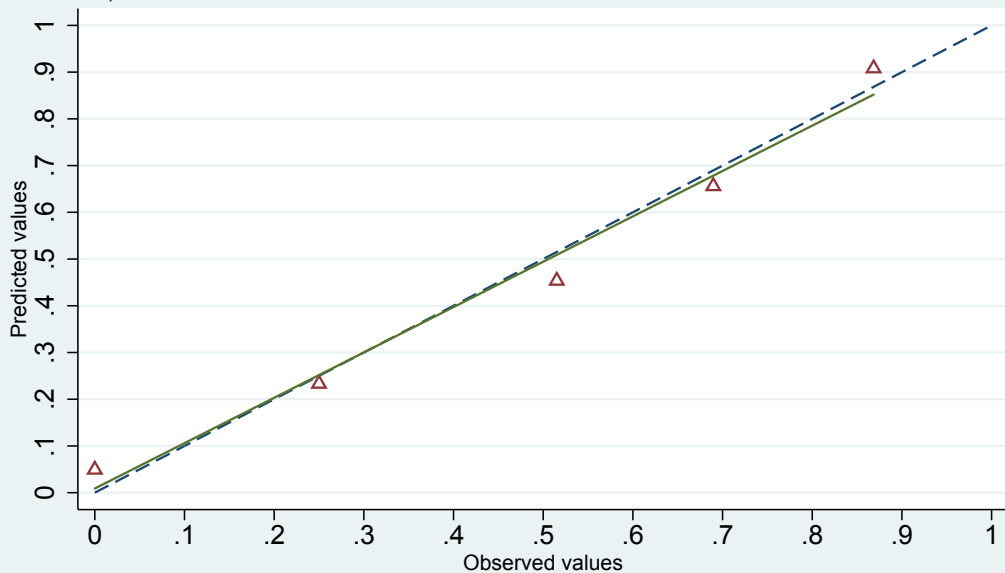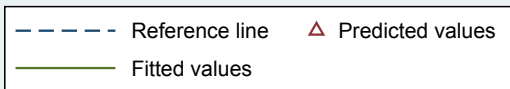

Supplement: Supplementary file 4 — Calibration plots for external validations at Thammasat University Hospital using different update methods. (ZIP 298 kb) [file 13017_2017_160_MOESM4_ESM.zip › Additional fig 3 G-M6.pdf]

A) Original model M0

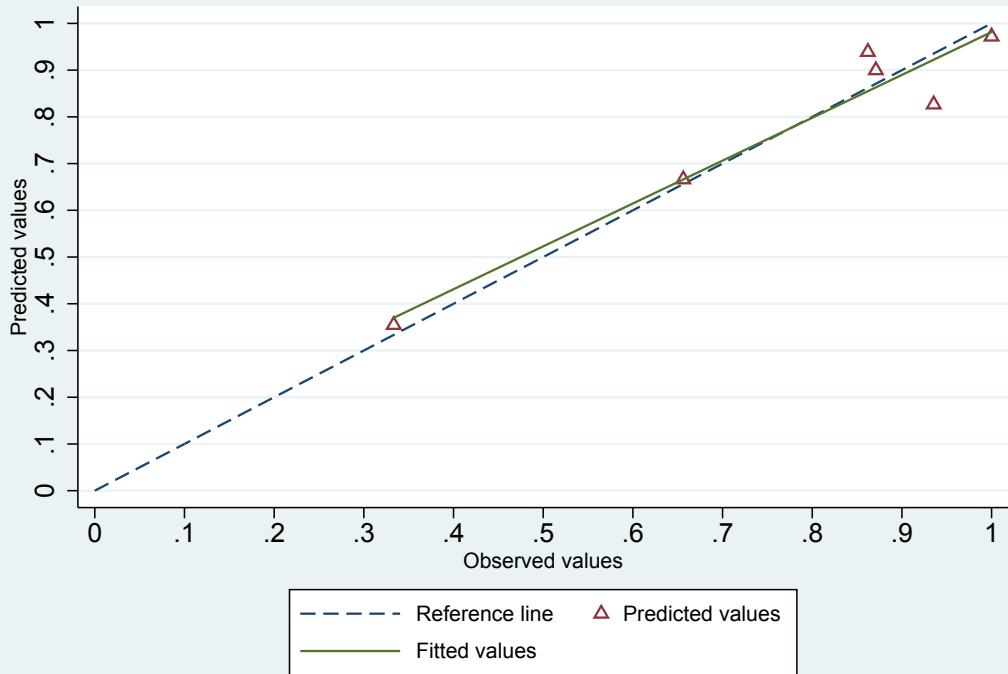

Supplement: Supplementary file 5 — Calibration plots for external validations at Chaiyapum Hospital using different update methods. (ZIP 298 kb) [file 13017_2017_160_MOESM5_ESM.zip › Additional fig 4 A-M0.pdf]

B) Re-calibration intercept M1

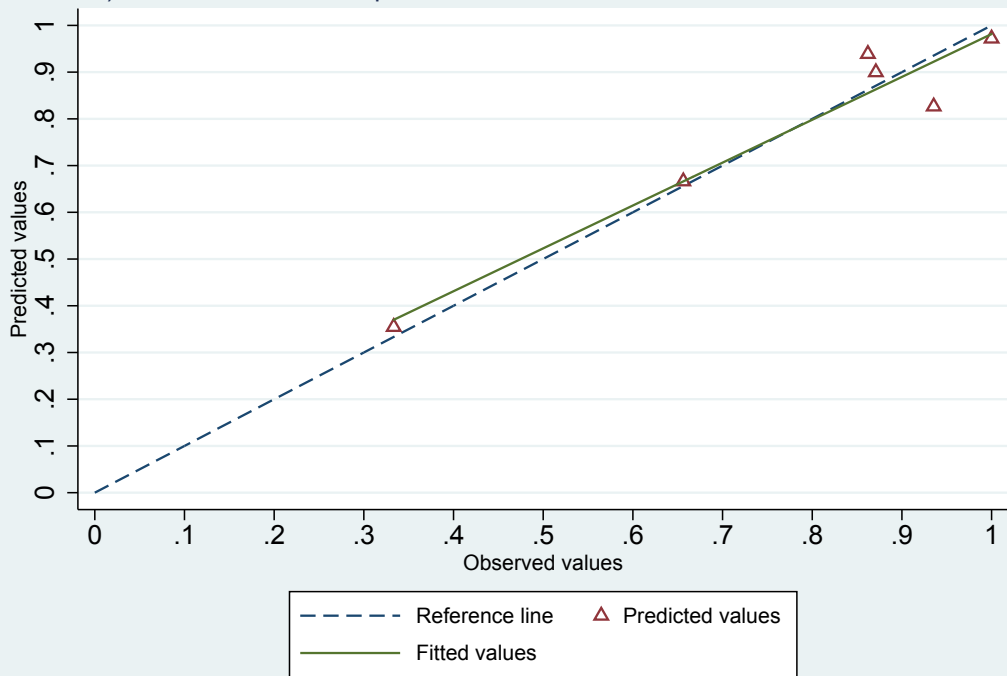

Supplement: Supplementary file 5 — Calibration plots for external validations at Chaiyapum Hospital using different update methods. (ZIP 298 kb) [file 13017_2017_160_MOESM5_ESM.zip › Additional fig 4 B-M1.pdf]

C) Re-calibration intercept and coefficient M2

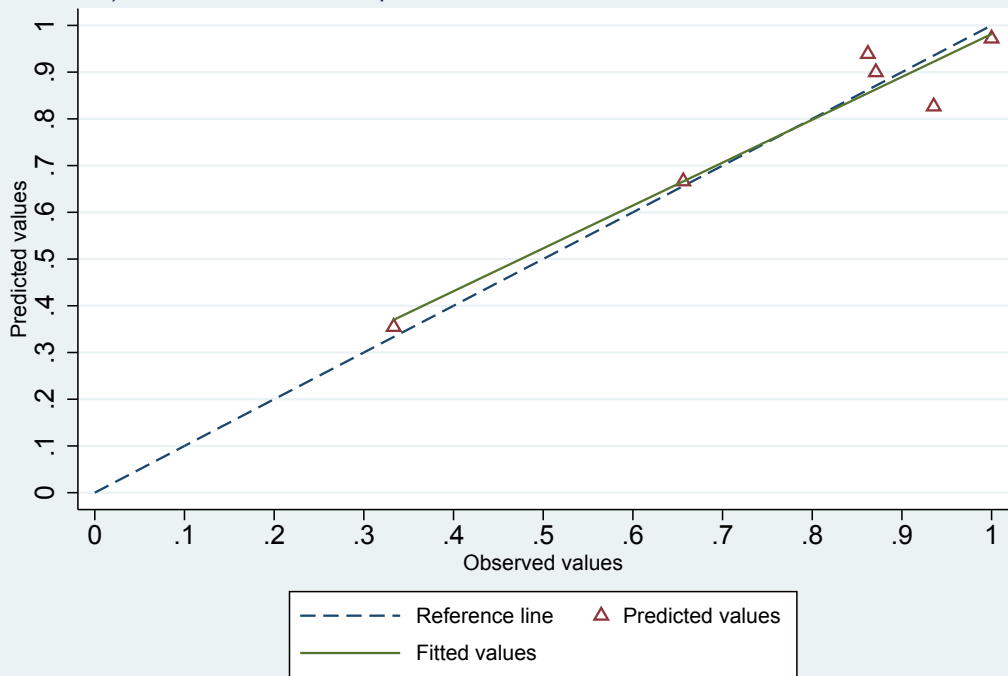

Supplement: Supplementary file 5 — Calibration plots for external validations at Chaiyapum Hospital using different update methods. (ZIP 298 kb) [file 13017_2017_160_MOESM5_ESM.zip › Additional fig 4 C-M2.pdf]

D) Revision model M3

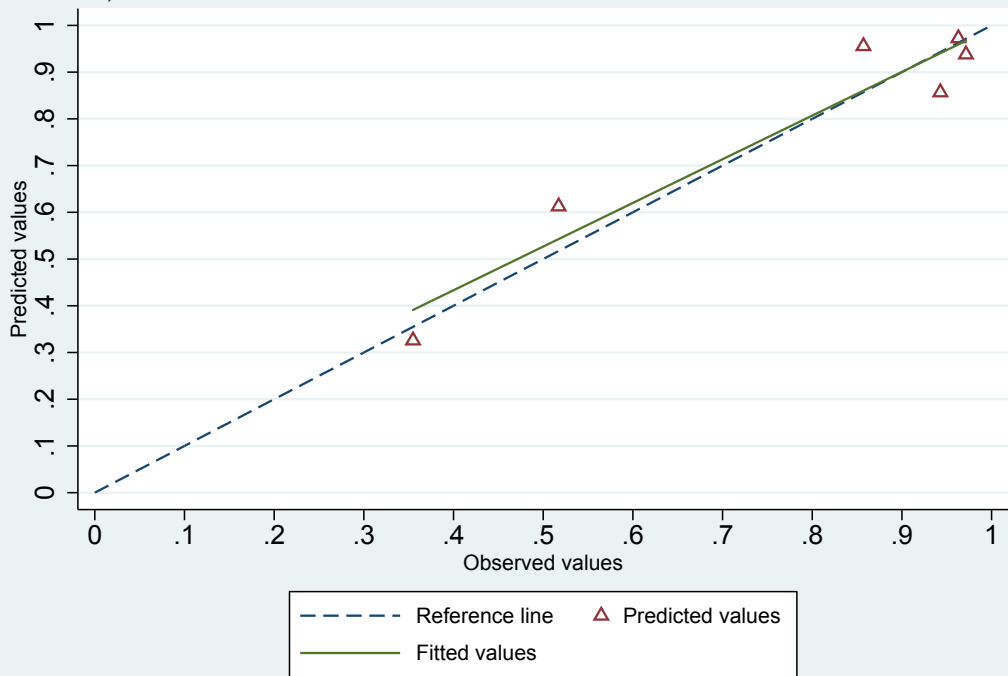

Supplement: Supplementary file 5 — Calibration plots for external validations at Chaiyapum Hospital using different update methods. (ZIP 298 kb) [file 13017_2017_160_MOESM5_ESM.zip › Additional fig 4 D-M3.pdf]

E) Revision model M4

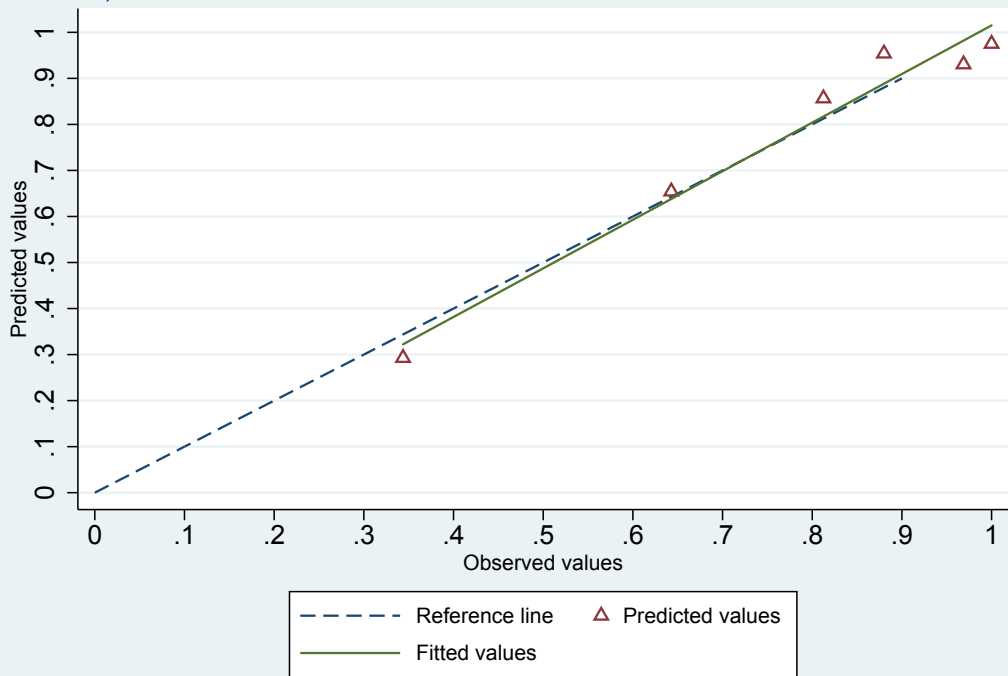

Supplement: Supplementary file 5 — Calibration plots for external validations at Chaiyapum Hospital using different update methods. (ZIP 298 kb) [file 13017_2017_160_MOESM5_ESM.zip › Additional fig 4 E-M4.pdf]

F) Revision model M5

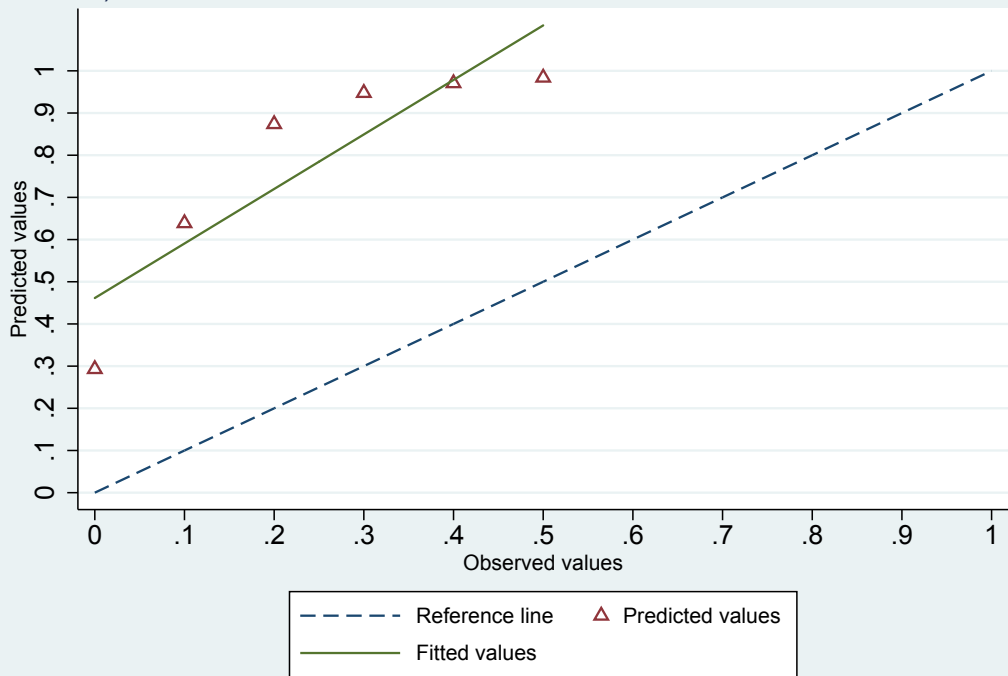

Supplement: Supplementary file 5 — Calibration plots for external validations at Chaiyapum Hospital using different update methods. (ZIP 298 kb) [file 13017_2017_160_MOESM5_ESM.zip › Additional fig 4 F-M5.pdf]

F) Revision model M6

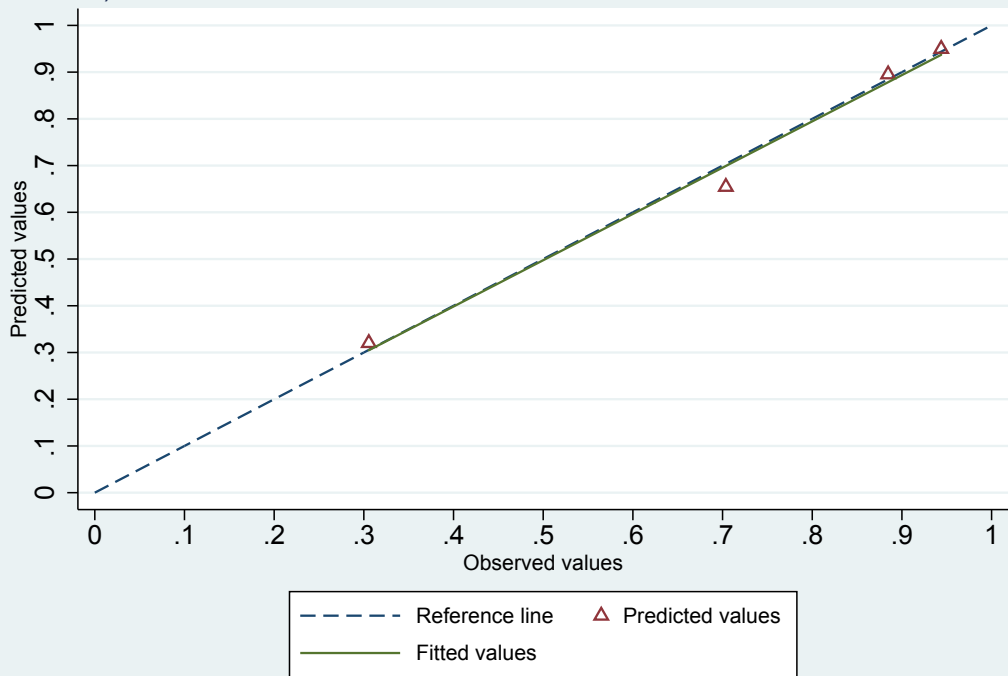

Supplement: Supplementary file 5 — Calibration plots for external validations at Chaiyapum Hospital using different update methods. (ZIP 298 kb) [file 13017_2017_160_MOESM5_ESM.zip › Additional fig 4 G-M6.pdf]
